# Supplementary material for: Are carbon emissions trading and green financial instruments synergistic? -Comprehensive quantitative research based on content analysis
Source: PLoS One. 2024 Mar 7;19(3):e0298601. doi: 10.1371/journal.pone.0298601 (PMC10919601; doi:10.1371/journal.pone.0298601)
Supplement: S1 Data — (ZIP) [file pone.0298601.s001.zip › Data/地级市Z_F工作报告.zip!/║■─╧/╒┼╝╥╜τ╩╨/└┤╫╘╣1⁄2╓┌║┼í╛┬φ┐╦╩2╛▌═°í┐_2013.docx]

一、过去四年工作回顾

过去的四年是极不寻常的四年。面对国际国内错综复杂的经济形势等一系列的严峻挑战和考验，我们始终高举中国特色社会主义伟大旗帜，坚持以邓小平理论、&ldquo;三个代表&rdquo;重要思想为指导，深入贯彻落实科学发展观，团结带领和紧密依靠全市各族人民，认真贯彻党中央、国务院的各项大政方针和省委、省政府的战略部署，全面落实市第五、第六次党代会和市五届人大会议确定的各项任务，解放思想，抢抓机遇，以改革促开放，以强基推转型，以发展惠民生，奋力实施&ldquo;三年有改观五年大变样&rdquo;工程，积极开启&ldquo;再干新五年再创新辉煌，奋力推进旅游产业大市向旅游经济强市跨越&rdquo;的新历程，取得了经济社会发展新的成就。

&mdash;&mdash;综合经济实力迈上新台阶。全市生产总值实现&ldquo;两个台级跳跃&rdquo;，2009年突破200亿元，比2008年增长13.7%；2012年突破300亿元，预计（下同）达到340亿元，比2008年增长65.6%，四年年均增长13.4%。财政总收入、公共财政预算收入在2008年的基础上实现&ldquo;两个翻了一番多&rdquo;，分别达到32亿元和22亿元，增长1.4倍和1.3倍。城镇居民人均可支配收入、农村居民人均纯收入实现&ldquo;两个两位数提升&rdquo;，分别达到15700元和4550元，年均增长10.4%和11.5%。

&mdash;&mdash;城市面貌发生显著变化。市城区和县城发展形成新格局，城镇化率由37.2%提高到41.1%。市城区建成区扩大三分之一，人均道路、公共绿地面积分别增长43.6%和22.1%，基础设施逐步配套，综合服务功能不断提升，吸附能力和辐射影响日益增强。国家旅游综合改革试点城市、中国最具海外影响力城市、世界旅游城市联合会会员城市、世界旅游可持续发展观测点、全国&ldquo;双拥&rdquo;模范城市等桂冠，赋予了我市发展新的魅力和张力。

&mdash;&mdash;旅游业转型升级取得实质性进展。多元化旅游格局开始形成，游客人均消费水平提高72.6%，购物和娱乐消费比重增加4.5个百分点。全市景点游客接待规模达到3500万人次，旅游总收入达到200亿元，分别是2008年的2.1倍和2.4倍。境外游客接待量实现翻番，达到220万人次，客源国和地区达到52个，韩国客源历久弥新，东南亚、欧美等国家和地区的游客达到54.4%。

&mdash;&mdash;文化产业异军突起，方兴未艾。&ldquo;国际乡村音乐周&rdquo;、&ldquo;天门狐仙?新刘海砍樵&rdquo;、&ldquo;张家界?魅力湘西&rdquo;等高端文化品牌，推动全市文化旅游跃居全省塔尖。文化与旅游融合发展，赋予张家界旅游新的内涵，&ldquo;白天赏美景、晚上看大戏&rdquo;成为游客在张家界新的消费理念。在演艺业的带动下，文博、娱乐休闲、影视、特色画等产业竞相发展，全市文化产业增加值占GDP比重达到8.1%，位居全省前列。

&mdash;&mdash;工业园区成为我市新型工业成形发展的重要依托。市经济开发区展现出工业新城风貌，慈利、桑植工业园跻身省级工业集中区，吸聚和承载能力明显增强。三个园区建成区达到7.8平方公里，入园企业达到94家，园区规模工业增加值达到 10 亿元，占全市规模工业增加值的比重由10.9%上升到15.8%，年创税收由1768万元增加到1.35亿元。

&mdash;&mdash;新型服务业态蓬勃发展。现代金融服务逐渐完善、产品不断创新，存贷款规模分别扩大1.3倍；现代物流业逐步壮大，产值年均增长20%以上；会展业快速成长，年营业收入达到2.7亿元；现代旅游地产正在兴起，酒店业发生了概念性的变化，一批五星级酒店和休闲度假设施拔地而起；现代信息产业快速发展，智慧旅游、智慧城市在创新中不断成长，全市服务业增加值年均增长14%。

&mdash;&mdash;&ldquo;四个农业&rdquo;带动城乡统筹发展。城市农业、旅游农业、品牌农业和生态农业呈现联点成片发展良好势头，城郊10万亩耕地退出传统农业种植，一大批村组成为&ldquo;四个农业&rdquo;规模化生产基地，市级以上农业龙头企业发展到80家，农民专业合作组织发展到421个，土地流转面积达到24万亩，蔬菜、生猪的市场自供能力分别上升20和15个百分点，耕地亩均产出、农业劳动生产率分别提高48.8%和63.8%，农业农村基础设施不断完善，新农村建设不断推进，农民收入年均增速高出城镇居民1.1百分点。

&mdash;&mdash;扶贫攻坚取得显著成效。全面完成第二阶段省级重点贫困村的建设扶贫任务，全面启动新一轮十年扶贫攻坚规划实施，&ldquo;基础先导、产业扶贫&rdquo;长效机制日趋完善。四年来，全市共投入6亿多元扶贫资金，基本解决了222个建设扶贫村产业开发、村民就医、学生上学等问题，实现了喝卫生水、走水泥路、用安全电的愿望，累计减少贫困人口 7.7万人。

&mdash;&mdash;民生保障体系基本形成。城镇零就业家庭保持动态清零，基本养老和基本医疗制度实现城乡全覆盖，城乡低保和农村五保实现应保尽保，义务教育阶段和中职学生实现免费入学，桑植、慈利两县农村义务教育学生纳入国家营养改善计划，参合农民乡镇住院起付线外费用全报销、高龄补贴、贫困大学新生救助、农村妇科病免费普查等制度相继建立，城镇中低收入家庭住房保障率达到94.8%，人民群众生活水平明显提高。

&mdash;&mdash;&ldquo;平安满意在张家界&rdquo;专项活动有效提升软实力。旅游市场环境、社会治安环境、政务服务环境持续改善，游客接待秩序、企业经营质量、市民文明素质明显改观，旅游的满意度和美誉度大幅提升，经济社会发展的吸引力和竞争力不断增强。

然而，即将过去的2012年，无论从全国全省看还是从我市看，应该是国际金融危机发生以来最为困难的一年。但是，我们在党中央、国务院和省委、省政府的正确领导下，适时地采取了一系列应对举措，&ldquo;保增长、惠民生、促和谐&rdquo;，基本实现全市稳中求进的总体目标。全市生产总值比上年增长11.6%；财政总收入、公共财政预算收入分别增长20%和21%；城镇居民人均可支配收入、农村居民人均纯收入分别增长11.2%和11%；固定资产投资、社会消费品零售总额、进出口总额分别增长17%、15.3%和38.5%；居民消费价格涨幅控制在2.5%以内；计划生育、安全生产工作进入全省先进行列，民生保障、节能减排等任务全面完成，社会事业全面进步，民主法制和精神文明建设进一步加强，社会大局保持和谐稳定。

各位代表，这四年，我们在市委的统一领导下，不断解放思想，锐意进取，砥砺前行，一步一个脚印地走来，创造了很多人民群众早已翘首期盼的发展业绩，办成了很多以前想办而难以办成的大事。是建市以来经济发展最快、固定资产投资最多、城乡面貌变化最大、人民群众感受最深得实惠最为明显的时期之一。

四年来，我们主要做了以下工作：

（一）科学谋划完善经济社会又好又快发展顶层设计。四年来，我们立足全市经济社会发展资源禀赋和现实基础，坚持以科学发展观为指导，解放思想，求真务实，坚持运用世界眼光、国际视野、战略思维，紧紧围绕建设世界旅游精品这个总目标不断创新发展思路、谋划完善顶层设计。一是全面建立了新一轮城乡统筹规划体系，修改了城市发展总体规划，编制了城市组团控制性详细规划和各专业专项规划；二是深入研究&ldquo;十一五&rdquo;发展实践，科学把握全市发展未来，按照&ldquo;以旅游为主导，新型工业强市，现代服务业兴市，加快城市建设，发展现代农业，促进扶贫攻坚&rdquo;的基本思路，精心编制了全市&ldquo;十二五&rdquo;发展规划，确立了&ldquo;把张家界建设成为功能齐全、交通便捷、产业发达、经济繁荣、人民富足、开放包容、文明和谐、具有优良生态环境和特有文化气质、绿色低碳、宜居宜游的国际风景旅游休闲度假城市&rdquo;的中心任务；三是在实现&ldquo;三年有改观五年大变样&rdquo;建设目标，广大人民群众对未来发展充满新期待的基础上，立足国家旅游综合改革试点、武陵山片区区域发展与扶贫攻坚试点、张家界国际航空口岸扩大开放三大平台，按照市第六次党代会提出的&ldquo;五化五建&rdquo;总体要求，确立了&ldquo;再干新五年再创新辉煌，奋力推进旅游产业大市向旅游经济强市跨越&rdquo;的部署安排，并编制了构建全面提升经济社会发展综合功能支撑体系的实施纲要。通过这些积极负责、不断深化的探索，全市经济社会发展的思路越来越清晰、着力点越来越明确、目标越来越实在、成效越来越显著。

（二）不断推进体制机制创新和开放合作。四年来，我们坚持把改革创新、扩大开放作为全市经济社会发展的第一推动力，真抓实干，力求突破。着力开展国家旅游综合改革试点，积极引导旅游企业集团化发展，全面启动旅游行业标准化建设，强力实施&ldquo;一诚通&rdquo;旅游目的地管理系统。着力深化行政管理体制改革，加快了财政、交通、水利、医药卫生等体制改革，文化体制改革被评为全国先进地区。着力实施投融资体制改革，继市经投集团公司、张股公司扩改之后，又积极组建了交通建设投资公司、水利建设投资公司、融资性担保公司、小额贷款公司、村镇银行、产权交易所等一批投融资平台；采用BT、BOT等多种战略融资合作方式实现融资81.2亿元。同时，通过搞活土地经营实现净收益10.7亿元。着力城市建设和管理体制机制创新，在全省率先实现了城乡绿化一体化管理，建立了城市规划审批、项目行政规费征收及减免、征地拆迁补偿安置、失地农民生活保障等一系列重大制度规范。着力加强市场体系建设，加快了国有企业和集体企业改革，完成了集体林权制度和农技推广服务体系改革，非公有制经济对全市GDP、税收的贡献率分别达到57%和64%。着力扩大对外开放、加快旅游国际化发展，相继引进4家外国航空公司参与荷花国际机场运营，建立了18个国际航点，台湾直航点获得认可；先后与13个外国城市建立国际友好合作关系，获得外国人来华访问邀请权；中旅总社等一批全国百强旅行社、铂尔曼等一批国际知名酒店品牌和华能集团等一批战略合作者进驻张家界。四年来，全市累计引进省外内资114.22亿元、外资1.62亿美元，分别是前四年的1.65倍和2.4倍。

（三）抢抓机遇下大力气加快城乡基础项目建设。四年来，我们奋力上项目、扩投资、强基础。累计完成市城区市政建设投资40.5亿元，新建和改造主次干道37条、小街小巷77条、供排水管网83.8公里、防洪堤20.58公里、公共绿地294.6公顷，并有效实施了主要街道&ldquo;穿衣戴帽&rdquo;综合整治工程。实现县城扩容提质投资8.6亿元，启动老观潭、蒋家坪新城开发，实施一批建制镇示范建设。累计完成交通建设投资65亿元，完成张花高速公路主体工程，建成张桑、龙阳、慈石、桑永等干线公路和市中心汽车站等一批客运站场，新建和改造农村公路2593公里，开工建设荷花机场扩建二期工程和张沅、桑鹤、慈茶等干线公路，扎实开展了黔张常铁路、张桑和慈安高速公路等重大项目前期工作。累计完成水利能源通信建设投资49亿元，新增水电装机22.51万千瓦，城乡电网规模实现翻番，农村电网改造面达到82%，管道燃气覆盖市县城区，电子政务外网、&ldquo;爱城市&middot;智慧张家界&rdquo;投入使用。累计完成农业基础建设投资35亿元，补充耕地2万亩，新增灌溉面积6.2万亩，建成高标准基本农田13.23万亩。

（四）大力调整产业结构，促进经济发展方式转变。四年来，我们坚持把调结构、促转型作为培育和壮大全市经济发展内生动力的关键举措来抓，坚持&ldquo;两轮驱动&rdquo;，通过改造提升和依托全市已有产业基础，保持较快的经济增长速度，加快发展步伐，不断缩小同全国全省和同类旅游城市的发展差距；通过调整经济结构，实现了一批产业转型升级，促进旅游经营由单一型向复合型转变、经济发展由粗放型向集约型转变，发展方式开始出现以基础设施建设拉动产业发展为主、同时以产业发展推进基础设施配套的双向促进、双向发展新格局。溪布街、天门洞体育生态公园等一批休闲度假项目与青和锦江、湘投阳光等一批高星级酒店的相继建成投产，贺龙体育中心、澧水风貌带、原生态绿景休闲园等转型项目的加快建设，&ldquo;张家界地貌&rdquo;获得国际权威认定，强力推动了我市旅游转型升级；大力培育发展工业经济，促进整体经济结构调整，市财政每年安排1500万元资金促进新兴产业发展，累计引导工业企业实现投资144亿元，工业增加值年均增长17.8 %；立足城乡统筹发展，促进农业增效、农民增收，加快推进&ldquo;四个农业&rdquo;发展，农业产业结构逐步实现全面调整，全市农业增加值年均增长4.5%；着力培育新型业态，加快发展现代服务业，积极落实扩内需、促消费政策，全市社会消费品零售总额年均增长16.6%。

（五）扎实推进社会事业发展和民生工程建设。筹资47.3亿元，全面完成145项省定实事和26项市定实事工程。累计投入就业和社保资金74.8亿元，新增城镇就业3.85万人、农村劳动力转移就业13万人、各类社会保险参保124.6万人次，新建和改建敬老院128所，建成各类保障性住房1.03万套，改造农村危房2.46万户，新增农村安全饮水人口41.35万人，职工最低工资、企业退休人员养老金、城乡低保补助、农村五保供养等标准提高一倍。累计投入社会发展资金88.8亿元，大力落实&ldquo;教育强市&rdquo;方略，实施了吉首大学张家界学院、张家界航院新校区等重大项目建设；新建和改扩建中小学校舍27.8万平方米，全面消除D级危房；大力发展基层卫生、文化、体育事业，完成县级医院、乡镇卫生院扩建任务，实现乡乡镇镇有综合文化站目标，社区体育健身设施全面实现免费开放；大力推进广播电视&ldquo;村村通&rdquo;工程，广播电视综合覆盖率达到96.5%；大力支持科技创新，专利授权和高新技术产业增加值年均分别增长37.8%和15.3%；大力加强监察、审计、人口和计划生育、妇女儿童、民族宗教、移民、统计、编制、档案、史志、残联等各项工作，加强国防教育和民兵预备役建设，促进了经济社会和谐发展。累计投入生态环保建设资金20亿元，建成一批污水处理厂和垃圾处理场，完成一批乡镇、村垃圾治理任务，实现节能减排目标，新增营造林100万亩，建成武陵源国家级生态示范区，全市森林覆盖率、城市空气质量优良率分别提高0.95和4.4个百分点。

（六）积极构建开放、务实、廉洁、诚信、高效的人民政府和法治政府。一是坚持把依法治市、民主决策、诚信办事贯彻到政府工作的各个环节，定期向人大、政协报告和通报工作，坚持重大决策集体决定、专家咨询、风险评估、合法性审查和重大事项社会公示、听证制度，建立并落实了邀请人大代表、政协委员和市民代表列席政府常务会议制度。二是认真贯彻《湖南省行政程序规定》和《湖南省政府服务规定》，制定完善和执行市政府工作规则、行政处罚裁量权基准，全面清理规范性文件和行政审批许可项目，积极推行&ldquo;一站式&rdquo;服务和重点岗位评议等工作制度，开通&ldquo;12345&rdquo;政府服务热线。三是深入开展&ldquo;机关效能建设&rdquo;和&ldquo;创先争优&rdquo;活动，落实政府绩效评估考核和行政问责，完善国土出让、工程招标、产权交易、政府采购、公务员招录、事业单位人员招聘等制度，严肃查处腐败案件，努力营造优良政务环境。四是以民生为大、以人民为重，狠抓安全生产和食品、药品、农产品安全，有效杜绝了重特大安全事故发生，安全事故死亡人数持续下降。五是狠抓社会综治与维稳工作，成功化解556件历史积案，保持了社会大局和谐稳定。

四年来的工作成就，标志着我们在建设世界旅游精品和富民强市的征程中迈出了新的坚实步伐，奠定了我们加快推进旅游产业大市向旅游经济强市跨越的良好基础。这些成果来之不易。这是党中央、国务院，省委、省政府正确领导的结果，是历届市委、市政府一届接着一届干、一届力求比一届干得更好的结果，是全市各族人民同心同德、共同努力和各方面热情关怀、大力支持的结果。在此，我代表市人民政府，向历届市委、市人大、市政府、市政协的老领导、老同志，向全市各族人民，向驻张部队、武警官兵和政法干警，向各民主党派、工商联、人民团体和各界人士，向所有关心支持张家界建设的国内外朋友、海外侨胞、台湾同胞、港澳同胞，表示衷心的感谢，并致以崇高的敬意！

四年丰富而生动的实践告诉我们，奋力推进旅游产业大市向旅游经济强市跨越，必须始终记取以下基本经验：

第一，必须不断解放思想，致力改革创新与开放合作的强力实施。解放思想就是机遇，解放思想就是项目，解放思想就是发展。只有坚决破除阻碍科学发展、跨越发展的思想观念和体制机制弊端，广泛吸纳先进发展理念和生产力要素，才能有效激发和聚集经济社会发展的动力和活力。

第二，必须坚持科学发展，致力加快发展与加快转型的互促共进。作为后发地区，实现经济社会又快又好发展，以加快发展促转型、以转型促进更大发展是我们的必然选择。只有在加快发展中实现转型、在加快转型中实现发展，才能推动全市经济社会科学发展、跨越发展。

第三，必须突出第一要务，致力强基础、调结构与扩投资、上项目。发展是硬道理，是解决一切问题的关键所在，任何时候都不能有丝毫动摇。只有准确把握基本市情，围绕基础配套、产业转型，聚精会神推进项目建设，千方百计扩大有效投资，以事业凝聚人心，以发展鼓舞人心，才能切实办好张家界的事情。

第四，必须落实城乡统筹，致力城市提升与农村发展的协调推进。建设小康张家界，美景在山，希望在城，难点在农村。只有突出城市建设，不断提升城市承载和辐射带动功能，统筹规划和建设城乡基础设施，实现城市市场、资本、技术、产业优势与农村资源优势的有机对接，才能落实&ldquo;以城带乡&rdquo;、&ldquo;以旅惠农&rdquo;和&ldquo;以工促农&rdquo;方略，切实加快&ldquo;三农&rdquo;发展。

第五，必须立足人民福祉，致力发展责任与民生责任的同步落实。做大&ldquo;蛋糕&rdquo;与分好&ldquo;蛋糕&rdquo;，都是人民政府义不容辞的责任。在大力发展经济的同时，必须坚持以人为本、勤政为民，统筹推进各项社会事业发展，着力解决人民群众最关心、最直接、最现实的利益问题。只有这样，才能让全体人民充分享受改革发展成果。

第六，必须严守发展底线，致力生态环境保护与经济社会发展的和谐统一。任何时候、任何情况下，都必须始终坚持&ldquo;生态立市、绿色发展&rdquo;和&ldquo;保护第一、永续利用&rdquo;的原则，实行最严格的环保政策，推进全方位的生态建设，不断增强可持续发展能力。只有保护&ldquo;绿水青山&rdquo;，才能赢得&ldquo;金山银山&rdquo;。

各位代表，过去的四年，我们取得了值得自豪的成绩，积累了弥足珍贵的经验，我们决不能妄自菲薄，但更不能妄自尊大。我们要清醒地看到，全市经济社会发展中基础差、底子薄、发展不快不优的基本市情还没有根本改变，发展水平与全国、全省相比仍然明显滞后，与全市各族人民的期盼还相距甚远。一是产业层级整体偏低，结构仍然不合理，发展内生动力不足；二是城乡基础设施还远不能适应产业发展和国际旅游目的地的要求；三是社会事业发展基础薄弱，统筹城乡发展任务艰巨，社会管理和服务与人民群众的期待还有较大差距；四是惯性思维、落后观念和僵化体制仍然是发展的最大障碍，少数公职人员服务意识不强，工作作风漂浮，甚至还存在腐败行为。

各位代表，在这里，还需要特别说明的是，2012年全市GDP增速虽然有望超过全省平均水平，但比年初预期目标低1.9个百分点，主要是固定资产投资、工业增加值等几项指标未能圆满实现。究其原因，主要是因为国际国内宏观经济环境复杂多变，后金融危机时期的深度影响由沿海向内地、由东部地区向西部地区蔓延的滞后性，对我省我市形成经济下行巨大压力；国家采取压缩调控政策，张花高速公路建设力度减弱，张桑高速公路、黔张常铁路等重大项目未能如期开工，投资在一定程度上形成了呆顿的局面；金融信贷增速趋缓，中小微小企业扶持政策难以落地，一些项目建设进度难以加快，一批工业企业生产严重不足；同时，解放思想不够、主观努力不够、工作落实不平衡，特别是征地拆迁面临多重压力，难以到位，也是其中的重要原因。

上述这些困难和问题，必须引起我们高度重视，要依靠我们进一步解放思想，创新思维，创新方式，依法治市，采取有效措施加以解决。

二、今后五年发展的基本思路和2013年的主要任务

各位代表，当前，我市已经站在一个新的阶段性发展层面。今后五年，我们的中心任务，就是紧紧围绕全面建成小康社会目标任务，加快实施全市经济社会发展&ldquo;十二五&rdquo;规划纲要，奋力推进旅游产业大市向旅游经济强市跨越。全市上下要在市委的统一领导下，高举中国特色社会主义伟大旗帜，以邓小平理论、&ldquo;三个代表&rdquo;重要思想和科学发展观为指导，认真贯彻党的十八大确定的大政方针，积极落实省委、省政府&ldquo;四化两型&rdquo;战略部署和市委&ldquo;五化五建&rdquo;总体要求，牢牢把握主题主线，&ldquo;再干新五年、再创新辉煌&rdquo;，着力增强创新驱动发展新动力，着力构建现代产业发展新体系，着力培育开放型经济发展新优势，着力形成城乡互促共进发展新格局，着力开创经济社会协调发展新局面，强化科学统筹，狠抓薄弱环节，加快推进世界旅游精品和武陵山片区中心城市建设，加快推进旅游产业大市向旅游经济强市跨越，到2020年同步全面建成小康社会。

实现&ldquo;同步全面建成小康社会&rdquo;目标任务，我们有基础、有条件，但也有难度、有压力。省统计局测算结果显示，2011年我市全面小康社会综合实现程度为78.4%，居全省第七位，但比全省水平低5.9个百分点，特别是经济发展、人均GDP、居民人均可支配收入、城乡居民收入比的实现程度分别仅为58.4%、46.5%、37.5%和44.9%。全市上下必须切实增强机遇意识、危机意识和责任意识，正视现实，坚定信心，凝心聚力，扎实奋斗。

今后五年，我们要牢牢把握解放思想这个发展关键，扎实推进科学发展、跨越发展。解放思想是科学发展观最鲜明的精神实质，是加快张家界发展的关键。要毫不动摇地扭住经济建设这个中心，勇于变革，不断创新，以更大的政治勇气和智慧，坚决破除一切妨碍科学发展的思想观念和体制机制弊端，坚持运用世界眼光，大胆吸收人类一切先进生产力要素和优秀文明成果，聚精会神搞建设，一心一意谋发展。

今后五年，我们要牢牢把握改革开放这个强大动力，扎实推进体制机制改革创新，提高开放合作水平。要深入实施国家旅游综合改革，努力在管理创新、基础配套、产业转型等方面取得&ldquo;先行先试&rdquo;成果；全面深化经济体制改革和机制创新，努力在更大程度更广范围发挥市场配置资源的基础性作用；更加有效地拓展开放合作，在国际间的交流、交往、交锋、交融中提升自身发展水平，在区域间合作共赢中抢夺发展机遇，在引商、引资、引智、引技等方面取得更大成效，努力打造活力之城、魅力之城、幸福之城。

今后五年，我们要牢牢把握扩大内需这个战略基点，扎实推进新型城镇化，促进城乡一体化发展。城镇化是扩大内需的最大潜力。要按照集约、智能、绿色、低碳、特色、文明的要求，加快形成布局合理、功能配套、运行协调、支撑有力的新型城镇化发展体系。大力提升中心城市集聚承载和辐射带动能力，大力增强县城和建制镇产业发展、吸纳就业、人口集聚功能，大力统筹城乡规划、基础建设和公共服务，落实完善户籍、土地、财税、住房、教育、社会保障等体制和政策，有序推进农业转移人口市民化。

今后五年，我们要牢牢把握调结构、转方式这个工作主线，扎实推进现代产业体系建设，加快形成新的经济增长方式。要全面深化产业结构战略性调整，坚持面向国际舞台推进旅游产业转型升级，坚持推动工业园区做大做强，坚持按照国际旅游目的地标准加快现代服务业发展，坚持以&ldquo;四个农业&rdquo;为重点引导现代农业发展，基本形成以旅游业为主导、多元产业支撑、发展特色鲜明、富有市场竞争力的新型产业体系，提高产业综合素质，实现尊重经济规律、有质量、有效益、可持续的发展。

今后五年，我们要牢牢把握文化这个发展新优势，扎实推进文化强市建设，不断壮大文化实力和竞争力。要着力加强社会主义核心价值体系建设，切实提高市民文明素质和社会文明程度。加快完善文化管理体制和文化生产经营机制，努力形成有利于创新创造的文化发展环境。加强重大公共文化工程和基层文化设施建设，完善公共文化服务体系。大力推进文化与旅游、科技深度融合，加快文化产业规模化、集约化、专业化发展，努力打造湖湘文化窗口和全国文化产业示范区。

今后五年，我们要牢牢把握科技进步这个重要支撑，扎实推进科技创新体系建设，全面提高经济社会发展科技水平。实现张家界经济社会又好又快发展必须依靠科技进步。要以创建新型旅游城市为目标，着力构建以企业为主体、市场为导向、产学研相结合的科技创新体系。以智慧旅游为重点，加快数字城市建设步伐。着力推进科技成果孵化平台建设，完善科技人才培养引进机制，健全科技服务体系，加强知识产权保护，切实提升科技实力，不断增强经济社会发展的内生动力和创造力。

今后五年，我们要牢牢把握以人为本这个核心立场，扎实推进社会事业全面发展，不断增进人民群众福祉。始终坚持把增加居民收入、提高人民群众幸福指数作为一切工作的出发点和归宿。全面加快各项社会事业发展，不断完善基本公共服务体系。大力实施教育强市建设，办好人民满意教育；加快发展医药卫生事业，为人民群众提供安全有效、方便价廉的公共卫生和基本医疗服务；多渠道创造就业机会，促进更高质量的就业；加大民生投入，不断完善全覆盖、保基本、多层次、可持续的社会保障体系。要突出薄弱环节，分类指导，切实加大扶贫攻坚力度，有效促进共同富裕。切实加强社会建设和社会管理创新，努力促进社会和谐进步。

今后五年，我们要牢牢把握生态文明这个长远大计，扎实推进&ldquo;两型&rdquo;社会建设，打造美丽家园。全面落实中央&ldquo;五位一体&rdquo;总体布局要求，把尊重自然、顺应自然、保护自然的生态文明理念，融入到经济建设、政治建设、文化建设、社会建设各个方面和全过程。着力加强生态文明制度建设，把资源消耗、环境损害、生态效益纳入经济社会发展评价体系；着力推进绿色发展、循环发展、低碳发展，全面实施环保十大工程建设，加快构建节约资源和保护环境的空间格局、产业结构、生产方式、生活方式；着力打造国家级森林城市和环保模范城市，积极争创全国生态文明实验区。

今后五年，我们要牢牢把握人民满意这个检验标准，扎实推进法治政府和服务型政府建设。加快行政管理体制改革和行政管理方式创新，促进政府职能向创造良好发展环境、提供优质公共服务、维护社会公平正义转变。加快依法治市进程，强化民主法制建设，着力提高领导干部运用民主法制的思维和方式深化改革、推动发展、维护稳定的能力。加快完善行政权力运行制约和监督机制，坚持科学决策、民主决策、依法决策，坚持从严治政，改进工作作风，密切联系群众，加强反腐倡廉，让权力在阳光下运行，致力营造干部清正、政府清廉、政治清明的良好局面。 各位代表，2013年是全面贯彻落实十八大精神的头一年，是奋力实施&ldquo;十二五&rdquo;发展规划的关键性一年，更是新一届政府履职的开局之年。纵观当前形势，世界经济进入深度转型调整期，&ldquo;竞争&rdquo;和&ldquo;壁垒&rdquo;交错依存，国际环境充满复杂性和不确定性，但世界多极化、经济全球化的趋势没有改变，和平与发展仍然是时代主题，全球合作正在向多层次全方位拓展。我国经济进入增长阶段转换期，原有竞争优势、增长动力逐渐削弱，新优势尚未形成，经济运行正处在寻求新平衡的过程之中，但是，中央稳增长的政策效应已逐步显现，经济运行筑底企稳。明年，中央继续实施积极财政政策和稳健货币政策，牢牢把握扩大内需这一战略基点，增强消费对经济增长的基础作用，发挥好投资对经济增长的关键作用，稳定完善强农惠农富农政策，加快调整产业结构，积极稳妥推进城镇化，全面深化经济体制改革，坚定不移扩大开放，加强民生保障和改善，为我们稳中求进提供了良多机遇。省委、省政府大力支持国家旅游综合改革和武陵山片区区域发展与扶贫攻坚，继续实施大湘西开发战略，扶持张家界发展的政策必将进一步得以落实，也给我们加快发展提供了更为广阔的空间。关键是看我们抓不抓得住、踩不踩得准。目前，我市正处在加快推进旅游国际化、工业化、城镇化、农业现代化和信息化的重要时期，虽然面临发展内生动力不足、要素瓶颈制约和思想观念滞后等诸多挑战，但同时也面临着很多新的发展机遇。武陵山片区区域发展与扶贫攻坚规划全面实施，黔张常铁路、张桑高速公路等一批重大项目建设已蓄势待发；国家旅游综合改革试点纵深推进，阻碍加快发展、加快转型的思想观念、体制机制性弊端和结构性难题必将有效破解；航空口岸扩大开放正朝着更高层次发展，对外开放和旅游国际化进程必将不断加快。特别是&ldquo;三年有改观五年大变样&rdquo;目标实现和&ldquo;再干新五年再创新辉煌&rdquo;工程开启，极大地鼓舞着全市各族人民追求更大更好更快发展的热情和斗志，使我们更加坚定了目标的自信、路径的自信和改革创新的自信。因此，新的一年，新的开局，全市上下要认真贯彻落实中央和省委经济工作会议精神，围绕主题主线，按照&ldquo;五化五建&rdquo;总体要求，进一步推动解放思想，进一步深化改革创新，进一步扩大开放合作，进一步注重质量和效益，致力扩内需、强基础、调结构、惠民生、促和谐，稳中求进，奋力推进张家界市新一轮的发展。

明年全市经济社会发展主要预期目标是：生产总值增长13%；其中一、二、三产业增加值分别增长4%、17%、14%；景点旅游人次和旅游总收入分别增长8%和15%，过夜游客增长10 %；规模工业增加值增长18%；固定资产投资增长20%；社会消费品零售总额增长18%；进出口总额增长15%；实际利用外资、内资分别增长10%；财政总收入增长17%，城镇居民人均可支配收入和农村居民人均纯收入分别增长11%；居民消费价格指数涨幅控制在4%左右；新增城镇就业1万人，城镇登记失业率控制在4%以内，人口自然增长率控制在7.5&permil;以内，亿元GDP安全生产事故死亡人数控制在0.23人以内；节能减排实现省定目标，生态环境质量进一步提高；社会事业全面进步，民主法制和精神文明建设进一步加强，社会大局保持和谐稳定。

这个预期目标安排与2012年相比，有6项指标作了调整。一是经济增速调低了0.5个百分点。主要是考虑两个方面的因素：一方面明年国际经济形势依然错综复杂、充满变数。由于受国际大环境的影响，国内经济发展仍然面临不少风险和挑战，我市经济发展明年的困难和压力仍然很大；另一方面从省到市州明年的经济增长预期都有所下调，我们提出13%的增速相比并不算低，这是考虑我们实现后发赶超、同步全面建成小康社会的任务还十分艰巨，必须保持经济持续快速增长。二是第一产业增加值增速调高了0.5个百分点。近四年来，随着&ldquo;四个农业&rdquo;的发展，我市第一产业增加值年均增长4.5%，农业结构调整成效不断显现，可以保持平稳较快发展。三是规模工业增加值增速调低了2个百分点。主要考虑：明年我国工业发展仍面临着需求不振、产能过剩等问题，我市工业缺乏大型项目支撑，形势难有大的改观，按照&ldquo;求真务实&rdquo;原则作了调整。四是城乡居民收入增速调高了1个百分点。到2020年实现城乡居民人均收入比2010年翻一番，是党的十八大的明确要求，也是我市同步全面建成小康社会的根本任务所在，我们必须更加有效地致力提高城乡居民收入水平。五是人口自然增长率调低了1.5个千分点。明年全省人口自然增长率控制目标是7&permil;，但考虑我市属少数民族地区、出生基数较大等因素，控制目标适当高于全省平均水平。六是亿元GDP安全生产事故死亡人数调低了0.02人。明年全省的控制目标是0.23人。近几年，我市安全生产基础工作明显加强，经过努力达到省控目标是应该而且可以做到的。

实现明年的各项预期目标，全市上下要凝心聚力，突出重点，真抓实干，着力抓好以下九个方面的工作：

（一）加快实施重大基础设施项目建设。大力实施以交通为重点的强基项目建设，力争黔张常铁路、张桑和慈安高速公路、绕城和环武陵源景区等干线公路、旅游航道开工建设，力争张花高速公路和张沅、桑鹤、慈茶等干线公路建成通车，加快荷花机场、航空口岸、城乡电网和通信网络等续建工程建设，扎实推进宜张、桑龙高速公路等项目前期工作。大力破解要素配置瓶颈，始终保持抓投资、扩投资、促投资的主动性，坚持把争取上级支持作为重要基点，继续努力争取国家和省里的项目布局和资金支持；进一步优化金融生态环境，强化政银企合作；做强交通建设投资公司、水利建设投资公司等新的融资平台；加大闲置土地清理处置力度，依法依规破解征地拆迁安置难题。大力推进项目招商，以诚招商，兑现承诺，形成以商招商的带动效应。大力落实保障服务，进一步完善市级领导、市直部门联系重点工程项目制度，强化项目调度和督导，加强施工环境治理，切实为项目建设保驾护航。

（二）大力拓展旅游产业发展纵深度，突出旅游转型升级，促进旅游国际化。支持武陵源国际旅游休闲度假区、天门山先导区、阳和国际旅游经济区建设，加快贺龙体育中心、原生态绿景休闲园和澧水风貌带建设，建成碧桂园、纳百利、禾田居等高星级酒店和杨家界索道、武陵源游客服务中心等旅游服务设施，启动实施武陵源核心景区扩容提质工程、张家界大峡谷二期工程和红二方面军长征纪念馆等项目建设。深化文化与旅游融合发展，建立旅游文化产业引导基金，推动魅力神歌集团上市，支持国际影视文化城建设。积极实施靠大联强战略，进一步加强与大型旅游集团、全国百强旅行社、国际知名品牌酒店、国内外航空公司的战略合作，加快旅游企业集约化、国际化发展。全面拓展客源市场，重点推进自助游、自驾游和会议旅游开发，进一步搞活国内市场；积极推进境外直航和落地签证，更加有效地拓展台湾、东南亚、欧美等境外市场；不断推进营销创新，办好第三届中国国际乡村音乐节，持续打造旅游兴奋点。坚持不懈开展&ldquo;平安满意在张家界&rdquo;专项活动，综合治理、惩防结合、标本兼治，不断擦亮&ldquo;平安满意&rdquo;品牌，全面提升张家界旅游的满意度和美誉度。

（三）加强城乡规划引导，切实加快城镇化建设步伐。按照城乡统筹发展的要求，全面实施城乡发展一体化规划，认真落实城乡规划法定图则管理，强力推进违法建设治理，不断规范建设秩序。加快城镇基础配套，以沙堤、且住岗、西溪坪为重点，集中连片推进城市功能区建设，启动武陵山大道、张清公路武陵源城区段改造和机场第二通道建设，建成溪北路等18条主次干道，实施回龙观公园二期等城市绿化景观工程。以蒋家坪、老观潭新区开发为龙头，加快推进县城扩容提质。积极探索小城镇建设投入机制，集中力量建设一批特色小城镇。加大城市管理力度，切实理顺市中心城区管理体制，积极推进相对集中行政处罚权和市政维护保洁市场化改革，充分发挥城市社区组织和机关单位作用，真正落实&ldquo;门前三包&rdquo;，实现&ldquo;人民城市人民管&rdquo;。统筹推进文明城市、卫生城市、森林城市、环保模范城市创建工作，不断提高城市序化、净化、绿化、美化水平。

（四）大力夯实工业园区平台，加快壮大工业经济发展实力。突出旅游商品产业园、软件园、港粤食品产业园、慈利工业大道建设，突出引进战略投资者，力争完成园区基础建设投资3.3亿元，新建标准厂房4.2万平方米，新增入园企业18家，园区工业增加值增长20%以上。大力促进工业项目落地投产，开工兴建科聚新材料、恒久高科、锦华药业等项目，力争华新水泥、金励矿业、经天光电、神舟医药等企业建成投产，力争页岩气项目进入勘探开发。大力增强科技创新能力，培育一批具有自主知识产权的企业，力争高新技术产业增加值增长20%以上。大力扶持小微企业加快成长，全面落实促进小微企业发展各项扶持政策，引导业主解放思想，靠大联强，推进机制、技术和管理创新，帮扶一批有资源优势、有科技含量、有市场前景的企业做大做强。

（五）继续深化农业结构调整，扎实推进农业现代化和新农村建设。在稳定粮油总产的基础上，实施农产品品牌战略，加快&ldquo;四个农业&rdquo;规模化、专业化、标准化发展，打造一批上规模、上档次、高效率的农业产业示范园区和农产品加工、销售龙头企业，着力提高蔬菜、水果、烤烟、茶叶、生猪、大鲵、中药材、花卉苗木、休闲农业的市场竞争力。统筹推进农村基础设施建设，加强农村公路建设和管理养护，加强农村土地综合整治，加强农田水利基本建设，继续推进农村电网改造、安全饮水和环境治理工程。鼓励发展新型农业生产经营模式，加强农民专业合作组织、农产品市场体系和农技、农机、农资、气象等服务体系建设。创新农村土地承包经营权流转机制，积极稳妥推进农村土地流转。盘活农村集体资产，培育发展村级集体经济。加强新农村示范片建设，力求取得更大成效。继续以桑植县为主战场，进一步完善部门包村和全社会共同参与的扶贫机制，加大扶贫投入，力争减少贫困人口3万人以上。

（六）围绕产业发展和扩大消费提高服务业发展水平。着力提升旅游服务，重点推进华天城、国际旅游商业城、家乐福购物广场等大型城市综合体建设，促进休闲度假、商务会展、文化娱乐、康体养生、旅游地产等新型服务业加快发展；着力加强生产性服务，加快建设物流园区和航空物流设施，建立健全技术产权交易市场，促进产业服务不断配套；着力强化开放服务，加大外商投资权益保护力度，扶持培育外贸企业成长，不断扩大服务贸易规模，加快开放型经济发展；着力改善居民消费服务，优化城乡商贸网点布局，加快城乡农贸市场改造升级，积极发展养老、托幼、家政等社区服务业，全面落实节能产品惠民政策，大力培育新的消费热点，不断扩大消费需求。

（七）加强以改善民生为重点的社会建设。不断提高民生保障水平，实施更加积极的就业政策，不断创新就业机制，努力提高就业质量。继续完善社会保障体系，努力探索旅游从业人员、失地农民养老保险统筹办法，完善城乡社会救助机制，新建各类保障性住房4500套，保障低收入群体基本生活；同时，加强民生政策宣传引导，鼓励广大群众依靠勤劳发家致富。不断完善基本公共服务体系，启动武陵山片区旅游医疗救治中心、张家界图书城和市技校搬迁建设；加快推进吉首大学张家界学院、张家界航院新校区、义务教育合格学校、中小学校舍安全工程建设，着力解决学前教育供给不足、城乡师资配备失衡、城市义务教育设施不配套等问题；加快改善农村和城市社区卫生医疗条件，深化医药卫生体制改革，全面落实国家基本药物制度；加强公共文化娱乐健身设施建设，实施广播电视&ldquo;户户通&rdquo;工程。不断加快各项社会事业进步，提高人口计划生育工作水平，开展第三次全国经济普查，加强国防、人防和民兵预备役建设，进一步做好妇女儿童工作，统筹推进民族宗教等各项社会事业发展。不断强化生态环保工作举措，加快推进生态市建设，积极推动武陵源核心景区生态移民工程，全面落实&ldquo;绿色湖南&rdquo;建设任务，确保实现节能减排年度目标。不断完善社会管理网络，强化安全生产和食品、药品、农产品质量监管，加强社会治安动态管控和信访维稳工作，保障人民群众安居乐业。2013年，市人民政府决定在确保完成省定为民办实事任务的同时，继续办好三件普惠性实事：一是为农村独生子女家庭年满50周岁及以上父母免费体检一次；二是为农村独生子女家庭危房改造户提供1万元补助；三是兴建老年人日间照料中心示范点10所。

（八）深入推进以旅游综合改革为重点的各项改革。深入推进旅游管理体制改革，力求在推进旅游服务标准化建设、建立健全旅游企业和从业人员资格准入与淘汰机制等方面取得更大进展，在建立大旅游工作格局、旅游综合执法机制、旅游佣金收授机制等方面实现突破，在理顺武陵源核心景区管理体制等方面迈出实质性步伐。深入推进市场化改革，充分发挥市场在资源配置中的基础性作用，不断强化资金、土地、技术、劳动力等要素保障能力；加快国企改制进程，盘活国有资产存量；放宽民间投资准入，不断激活民间资本。深入推进金融产品创新和新型金融体系建设，积极引进股份制商业银行和证劵、保险机构，加快组建县域农村商业银行、村镇银行、小额贷款公司、融资性担保公司等新型金融机构，创立私募股权投资基金、创业基金，积极推动优质企业上市、债券发行、股权融资。深入推进社会管理体制改革，加快构建科学有效的社会管理体制，加强基层社会管理和服务体系建设；健全社会治安防控体系，提升防、管、控、打能力，打造平安城市；创新交通管理体制机制，启动交通管理模范城创建工作。深入推进行政管理体制改革，加快政企分开、政资分开、政事分开、政社分开步伐，切实转变政府职能；加快财政体制改革，进一步理顺和完善市、区、县及经济开发区的财政体制；加快完善行政审批制度，牢固树立企业至上、服务第一理念，致力创造零障碍、低成本、高效率的政务环境。

（九）全面构建法治政府和服务型政府，努力转变工作作风。坚持依法治市、依法行政，切实加强民主法制建设，全面推进依法治理，严格执行重大事项议事规则和决策程序，自觉接受人大及其常委会的法律监督和工作监督，自觉接受人民政协、民主党派、工商联和社会各方面的监督，加快行政决策和行政管理的科学化、民主化、法制化进程；坚持真抓实干、勤政奉公，加强机关效能建设，全面推行服务承诺制和限时办结制，强化绩效评估考核，崇尚实干，力戒空谈，采取过硬措施，精简会议，改进会风，厉行节约，治庸、治懒、治散、治奢、治腐，促使各级干部在其位、尽其责，干干净净、朝气蓬勃谋其事，坚决杜绝软弱漂浮、抓而不实、推诿扯皮等现象。坚持转变观念，开拓进取，开展新一轮解放思想大讨论，在解放思想中统一思想、凝聚共识，真正形成团结协作、共谋发展的工作格局；加强实际工作能力锻造，引导各级干部自觉提高驾驭市场经济的能力、开展新形势下群众工作的能力和推动工作落实的能力，更加奋发有为地干事创业。坚持密切联系群众、真诚服务群众，带头落实中央关于改进工作作风、密切联系群众的八项规定，深入基层，深入群众，真心实意为人民群众办实事、解难事，以实际行动赢得群众支持；带头向群众学习，向实践学习，善于凝聚群众的热情和智慧破解难题、化解矛盾、落实工作。

各位代表，我们面临的任务艰巨而繁重，肩负的责任重大而光荣。让我们在省委、省政府和市委的统一领导下，坚定必胜信心，勇敢迎接挑战，扎实工作，锐意进取，不断创造无愧于时代、无愧于历史、无愧于党和人民的新业绩，为实现旅游产业大市向旅游经济强市跨越、加快全面建成小康社会步伐而努力奋斗！
